# Methodology for forecasting electricity consumption by Grey and Vector autoregressive models

**DOI:** 10.1016/j.mex.2021.101296

**Published:** 2021-03-04

**Authors:** Serge Guefano, Jean Gaston Tamba, Tchitile Emmanuel Wilfried Azong, Louis Monkam

**Affiliations:** University of Douala, University Institute of Technology of Douala, Cameroon

**Keywords:** Forecast, Electricity consumption, Grey model, VAR model, hybrid model GM(1,1)-VAR(1)

## Abstract

Forecasting energy demand in general, and electricity demand in particular, requires the developing reliable forecasting tools that can be used to monitor the evolution of consumers’ energy needs more accurately. The proposed new hybrid GM(1,1)-VAR(1) model is meant for that purpose. The latter is based on the Grey and Vector autoregressive approaches, and makes it possible to predict future demand, by taking into account economic and demographic determinants with an exponential growth trend. With an associated APE of 1.5, a MAPE of 1.628%, and an RMSE of 15.42, this new model thus presents better accuracy indicators than hybrid models of the same nature. Also, it proves to be as accurate as some recent hybrid artificial intelligence models. The model is thus a reliable forecasting tool that can be used to monitor the evolution of energy demand.

• The Grey and Vector autoregressive models are coupled to improve their accuracy.
• Five economic and demographic parameters are included in the new hybrid model.
• This new model is a reliable forecasting tool for assessing energy demand.

## Highlights

• The Grey and Vector autoregressive models are coupled to improve their accuracy.
• Five economic and demographic parameters are included in the new hybrid model.
• This new model is a reliable forecasting tool for assessing energy demand.

## Graphical abstract

**Figure:**
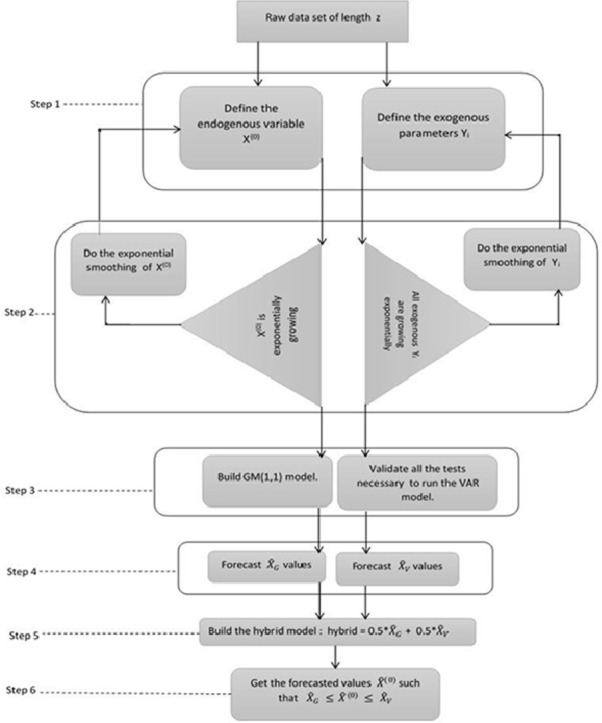

Specifications Table

**Table:** 

Subject Area	Energy
More specific subject area	*Electricity demand forecast*
Method name	New hybrid GM(1,1)-VAR(1) model.
Name and reference of original method	*WANG, Zheng-Xin, LI, Qin, et PEI, Ling-Ling. A seasonal GM (1,1) model for forecasting the electricity consumption of the primary economic sectors. Energy, 2018, vol. 154, p. 522–534.XU, Ning, DANG, Yaoguo, et GONG, Yande. Novel grey prediction model with nonlinear optimized time response method for forecasting of electricity consumption in China. Energy, 2017, vol. 118, p. 473–480.YUAN, Chaoqing, LIU, Sifeng, et FANG, Zhigeng. Comparison of China's primary energy consumption forecasting by using ARIMA (the autoregressive integrated moving average) model and GM (1,1) model. Energy, 2016, vol. 100, p. 384–390.DING, Song, HIPEL, Keith W., et DANG, Yao-guo. Forecasting China's electricity consumption using a new grey prediction model. Energy, 2018, vol. 149, p. 314–328.YE, Jing, DANG, Yaoguo, DING, Song,* et al. *A novel energy consumption forecasting model combining an optimized DGM (1,1) model with interval grey numbers. Journal of Cleaner Production, 2019, vol. 229, p. 256–267.XU, Weijun, GU, Ren, LIU, Youzhu,* et al. *Forecasting energy consumption using a new GM–ARMA model based on HP filter: The case of Guangdong Province of China. Economic Modelling, 2015, vol. 45, p. 127–135.*
Resource availability	*-Latex file*

## Method details

In this section, we have set to describe the various steps that are require for modeling and forecasting electricity consumption from each of the previous models.

## The Grey model

All the steps aiming at developing the GM(1,1) Grey model are described as follows:

- **Step one**: Exponential smoothing of raw data.

It is worth noting that forecasts based on Grey models are satisfactory when raw data show an increasing exponential trend. In case the observed trend among basic data is not exponential, Yuan C. et al. [1] suggest to use an Exponential smoothing of raw data, and to only work with actual data whose relative gap is less than 0.5. In this paper, that stage is a sinequanon condition to executing the Grey GM (1,1) model.

All other stages required for the implementation of this model are described as follows [2]:

- **Step two**: raw data processing.

Let X^(0)^ be the raw data set defined by *X*^(0)^ = (*x*^(0)^(1); *x*^(0)^(2); *…; x*^(0)^(*n*)) where *x*^(0)^(*i*) *≥* 0 and

*i* = 1*,* 2*, …, n*. We further define the *X*^(1)^ series known as 1-AGO sequence as:

$$X^{\left(1\right)}=\left(x^{\left(1\right)}\left(1\right);x^{\left(1\right)}\left(2\right);...;x^{\left(1\right)}\left(n\right)\right)$$

with $x^{\left(1\right)}\left(k\right)=\sum_{i=1}^{k}x^{\left(0\right)}\left(i\right)$and *k* = 1,2,…,n.

- **Step three**: estimate of model parameters *a* and *b*. This is done by considering the first form of the GM(1,1) model defined by Eq. (1):
  (1) $$x^{\left(0\right)}\left(k\right)+az^{\left(1\right)}\left(k\right)=b$$

where *a* and *b* are respectively the parameters of development and control of the model, and *z*^(1)^(*k*) the background sequence of the model given by the Eq. (2):

(2) $$z^{\left(1\right)}\left(k\right)=0.5x^{\left(1\right)}\left(k\right)+0.5x^{\left(1\right)}\left(k-1\right)$$

where *k* = 2*,* 3*, …, n*. Considering the values of *k*, we obtain the system (*s*_0_)

$$\left(s_{0}\right)=\left\{ \begin{array}{c} z^{\left(1\right)}\left(2\right)=0.5x^{\left(1\right)}\left(2\right)+0.5x^{\left(1\right)}\left(1\right)\\ z^{\left(1\right)}\left(3\right)=0.5x^{\left(1\right)}\left(3\right)+0.5x^{\left(1\right)}\left(2\right)\\ z^{\left(1\right)}\left(4\right)=0.5x^{\left(1\right)}\left(4\right)+0.5x^{\left(1\right)}\left(3\right)\\ .\\ .\\ .\\ z^{\left(1\right)}\left(n\right)=0.5x^{\left(1\right)}\left(n\right)+0.5x^{\left(1\right)}\left(n-1\right) \end{array}\right.$$

By introducing each equation of the previous system into Eq. (1), the following system (S) is obtained

$$\left(s\right)=\left\{ \begin{array}{c} x^{\left(0\right)}\left(2\right)=-az^{\left(1\right)}\left(2\right)+b\\ x^{\left(0\right)}\left(3\right)=-az^{\left(1\right)}\left(3\right)+b\\ .\\ .\\ .\\ x^{\left(0\right)}\left(n\right)=-az^{\left(1\right)}\left(n\right)+b \end{array}\right.$$

The system (S) then takes the matrix form *Y* = Br where:

$$Y=\left[\begin{array}{c} x^{\left(0\right)}\left(2\right)\\ x^{\left(0\right)}\left(3\right)\\ .\\ .\\ .\\ x^{\left(0\right)}\left(n\right) \end{array}\right],\;B=\left[\begin{array}{c} -z^{\left(1\right)}\left(2\right)...1\\ -z^{\left(1\right)}\left(3\right)...1\\ .\\ .\\ .\\ -z^{\left(1\right)}\left(n\right)...1 \end{array}\right],\;r=\left[a,b\right]$$

Thus, with ordinary least squares method, parameters a and b of the following matrix are estimated:

$$\hat{r}={\left[\hat{a},\hat{b}\right]}^{T}\;as:\;\hat{r}={\left(B^{T}B\right)}^{-1}B^{T}Y$$

- **Step four**: search for the temporal response function.

For this purpose, we consider the differential equation of the Grey model defined by Eq. (5).

(5) $$\frac{dx^{\left(1\right)}\left(t\right)}{dt}+ax^{\left(1\right)}\left(t\right)=b$$

Solving the latter equation yields the time response function described by Eq. (6).

(6) $${\hat{x}}^{\left(1\right)}\left(t\right)=Ce^{\left(-\hat{a}t\right)}+\hat{b}\;/\;\hat{a}$$

where *C* is a constant that depends on the initially chosen condition. With *x*ˆ^(1)^(*t*)*|_t_* _=_ _1_ = *x*ˆ^(1)^(1) = *x*^(0)^(1), we get to $C=\left(x^{\left(0\right)}\left(1\right)-\hat{b}\;/\;\hat{a}\right)e^{\hat{a}t}$, and the Eq. (6) appears as shown in relation (7).

(7) $${\hat{x}}^{\left(1\right)}=\left(x^{\left(0\right)}\left(1\right)-\hat{b}\;/\;\hat{a}\right)e^{-\left(t-1\right)\hat{a}}+\hat{b}\;/\;\hat{a}$$

With *t* = 2,3,…,n.

The IAGO sequence from the relation ${\hat{x}}^{\left(0\right)}\left(t\right)={\hat{x}}^{\left(1\right)}\left(t\right)-{\hat{x}}^{\left(1\right)}\left(t-1\right)$, allows to obtain the response function restored in equation (8).

(8) $${\hat{x}}^{\left(0\right)}\left(t\right)=\left(x^{\left(0\right)}\left(1\right)-\hat{b}\;/\;\hat{a}\right)\left(1-e^{\hat{a}}\right)e^{-\left(t-1\right)\hat{a}}$$

With *t* = 2, 3, …, n, *n* + 1, *n* + 2,…

Consequently, for *t* = 2*,* 3*, …, n, x*ˆ^(0)^(*t*) are the filtered values and for *t* = *n* + 1*, n* + 2*, …,x*ˆ^(0)^(*t*) are the forecasted values.

### Forecasting with Grey model

The specificity of the Grey GM(1,1) model is that it provides fairly accurate forecasts based on a small range of actual data. For this reason, the rolling mechanism makes use of the most recent data to forecast future consumption values with greater accuracy [3].

For a series (*x*^(0)^(1); *x*^(0)^(2); *…; x*^(0)^(*n*)) of n raw data, let's assume that *z* data are used to construct the GM(1,1) model, and *q* values are estimated to test the model's forecasting capacities. The procedure for running GM (1,1) is summarized in three steps as follows:

**Step one**: we consider sequence (*x*^(0)^(1); *x*^(0)^(2); *…; x*^(0)^(*z*)) to calibrate the model by determining values *a* and *b* of the coefficients of the model corresponding to this series. Then, values (*x*ˆ^(0)^(*z* + 1)*, x*ˆ^(0)^(*z* + 2)*, …, x*ˆ^(0)^(*z* + *q*)) are estimated.
**Step two**: Consider a new series of raw data of *z* length to recalibrate the model by calculating parameters *a* and *b*. Then, (*x*ˆ^(0)^(*z* + *q* + 1)*, x*ˆ^(0)^(*z* + *q* + 2)*, …, x*ˆ^(0)^(*z* + 2*q*)) values are estimated.
**Step three**: repeat step 2 until the desired forecast horizon is reached.

### Assessment of the model accuracy

Five descriptive statistics are used in this paper to analyze the reliability and the accuracy of each model. These are the APE (Absolute Percentage Error), the MAPE (Mean Absolute Percentage Error), the MSE (Mean Square Error), The RMSE (Root Mean Square Error) [3] and Nash-Sutcliffe (NS)’ statistics. The APE and MAPE provide information on the forecast capacity of the model. The MSE and the RMSE of the model, provide information on the fidelity and righteousness of the model.

The more the RMSE value is closer to zero, the better the formulated model. The NS statistic is a performance indicator which helps estimating the capacity of a model to reproduce an observed behavior. For a model to be judged satisfactory, we must have NS≥0.7 that is, the observed models must be coherent. Those indicators are calculated based on Eqs. (9)–(13).

(9) $$APE=\left[\frac{\left|{\hat{x}}^{\left(0\right)}\left(k\right)-{\hat{x}}^{\left(0\right)}\left(k\right)\right|}{x^{\left(0\right)}\left(k\right)}\right]*100$$

(10) $$MAPE=\frac{1}{n-1}\sum_{k=2}^{n}APE(k)$$

Table 1 gives the MAPE assessment criteria for a statistical series.

(11) $$MSE=\frac{1}{n-1}\sum_{k=2}^{n}{\left({\hat{x}}^{\left(0\right)}\left(k\right)-x^{\left(0\right)}\left(k\right)\right)}^{2}$$

(12) $$RMSE=\sqrt{MSE}$$

(13) $$NS=1-\frac{MSE}{\sigma^{2}}$$

where *x*^(0)^(*k*) are the actual values, *x*ˆ^(0)^(*k*) the forecast values, and *σ*^2^ the variance associated to the parameter *X*^(0)^. Other statistics are used in this work for the analysis of retained parameters. These are namely the correlation coefficient *ρ*, used to capture the link between two parameters, and the Average Annual Growth Rate (AAGR) which shows the evolution percentage of a parameter over consecutive years.

**Table 1:** MAPE criteria [3].

MAPE (%)	Forecasting capacity
<10	Excellent
10–20	Good
20–50	Reasonable
≥50	bad

## The Vector autoregressive model

The multilinear Vector Autoregressive (VAR(p)) model formulated in 1980 by Christopher Sims is a generalization of the ARMA models formulated by Box and Jenkins in 1978 [4]. Here, the electricity consumption known as *x*(*t*) at a *t* date is specified as a function of past values *x* (*t − i*) and residuals

(14) $$\begin{array}{c} \varepsilon \left(t-i\right)\\ x\left(t\right)=\sum_{i=1}^{p}\alpha_{i}x\left(t-i\right)+\sum_{i=1}^{q}\beta_{i}\varepsilon \left(t-i\right) \end{array}$$

This equation is a linear one, where electricity consumption solely depends on its previous values. Christopher Sims generalizes the latter relationship in the form generated by 15, whereby the consumption x(t) can depend not only on the previous values x(t - i), but also on some exogenous parameters including GDP $y_{p}\left(t-i\right)$, GDP per capita $y_{ph}\left(t-i\right)$, population $y_{po}\left(t-i\right)$, number of households $y_{a}\left(t-i\right)$, and number of subscribers . In this case, Eq. (14) takes form 15.

(15) $$\begin{array}{ccc} x(t) & = & a_{1}^{0}+\sum_{i=1}^{z}\left[a_{1i}^{1}x\left(t-i\right)+a_{1i}^{2}y_{p}\left(t-i\right)+a_{1i}^{3}y_{ph}\left(t-i\right)\right]\\ & & +\;\sum_{i=1}^{z}\left[a_{1i}^{4}y_{po}\left(t-i\right)+a_{1i}^{5}y_{m}\left(t-i\right)+a_{1i}^{6}y_{a}\left(t-i\right)\right]+\mu \left(t\right) \end{array}$$

In this last equation $a_{1i}^{j}$ for *j ∈* [1*,*6] are coefficients of the model, $a_{0}^{1}$ is the constant term and $\mu_{t}$ is the white noise error term . Provided the optimal lag number p retained by the statistic tests is 1, the electricity consumption on the t date may depend on all variables lagged by one period. From that perspective, Eq. (15) takes form 16.

(16) $$\begin{array}{ccc} x(t) & = & a_{1}^{0}+a_{11}^{1}x\left(t-1\right)+a_{11}^{2}y_{p}\left(t-1\right)+a_{11}^{3}y_{ph}\left(t-1\right)\\ & & +\;a_{11}^{4}y_{po}\left(t-1\right)+a_{11}^{5}y_{m}\left(t-1\right)+a_{11}^{6}y_{a}\left(t-1\right)+\mu \left(t\right) \end{array}$$

This result is an equation where parameters are chronological variables which often present a non-stationary evolution. Such chronological variables in a forecasting model, imply interdependence among errors’ terms, as well as the bias of forecasting results. In that case, one or several filters at differences are necessary in order to make all exogenous and endogenous variables stationary [5]. If only one filter is needed to stabilize each parameter, then the previous equation takes form 17.

(17) $$\begin{array}{ccc} \Delta x(t) & = & a_{1}^{0}+a_{11}^{1}\Delta x\left(t-1\right)+a_{11}^{2}\Delta y_{p}\left(t-1\right)+a_{11}^{3}\Delta y_{ph}\left(t-1\right)\\ & & +\;a_{11}^{4}\Delta y_{po}\left(t-1\right)+a_{11}^{5}\Delta y_{m}\left(t-1\right)+a_{11}^{6}\Delta y_{a}\left(t-1\right)+\mu \left(t\right) \end{array}$$

Such a model must then be statistically validated before attempting any forecast.

### Validation of the Vector autoregressive model

Once the coefficients $a_{1i}^{j}$ have been estimated, we perform three statistical tests of greater significance, in order to validate the formulated model. This is the unit root test based on Dickey Fuller Augmented (ADF) and Phillips-Perron (PP) statistics, the test on the normality of residuals using the Jarque-Bera statistic as defined by Eq. (18), and the model stability testing using the cumulative sum of the recursive residuals *S*(*t*) and the cumulative sum of the squares of the recursive residuals S’(t) defined by formulas (a) and (b) of Eq. (19).

(18) $$JB=n\;/\;6\beta_{1}+n\;/\;24{\left(\beta_{2}-3\right)}^{2}$$

$\beta_{1}$ and $\beta_{2}$ are respectively coefficients of Skewness and Kurtosis.

(19) $$\left\{ \begin{array}{c} S\left(t\right)=\left(n-k\right)\frac{\sum_{j=k+1}^{t}{\tilde{\varepsilon }}_{j}}{\sum_{j=k+1}^{n}{\tilde{\varepsilon }}_{j}}\left(a\right)\\ S^{\prime }\left(t\right)=\frac{\sum_{j=k+1}^{t}{{\tilde{\varepsilon }}^{2}}_{t}}{\sum_{j=k+1}^{n}{\tilde{\varepsilon }}_{j}^{2}}\left(b\right) \end{array}\right.$$

In this equation, *µ*˜*_j_* is the error term, and represents the difference between the actual model and the estimated model.

In fact, Provided we have level stationary variables at the end of our tests, they are designated I(0). If on the other hand, others are stationary after one or n first-differences, they are designated I(1) or I(n). Therefore, VAR(p) modelling is only possible if all observables are stable at the same order.

The formulated model is statistically stable when the evolution of the sum of the error terms is within the confidence interval *β* = *±*95% at the critical threshold of *α* = 5%. Provided the Jarque-Berra statistics are less than 5*.*99 with a *P-value* greater than 5%, residuals are independent and normally distributed within the model. They therefore constitute a white Gaussian noise [5].

### Forecasting with the Vector autoregressive model

In a situation where the model parameters are stationary after a first difference, the forecasting approach in difference can be adopted in order to sequentially calculate future values of electricity consumption. This is done using the relationship given by Eq. (20), where *x*ˆ*_t_* is the estimated value of consumption on the *t* date, *x_t−_*_1_ is the revealed value of the consumption on *t-*1 date, and ∆*x* is the difference of consumption between years *t* and, t-1 and estimated by the software

(20) $$\Delta x_{t}={\hat{x}}_{t}-x_{t-1}$$

The error associated with each estimate is digitally estimated from Eqs. (9) and (13).

### The hybrid model

The construction of the structure of the GM(1,1)-VAR(p) hybrid model is conditioned by the characteristics of the respective residuals of each of the models [1]. If their residuals are mutually opposite in signs, and if those of the VAR(p) model are normally distributed, then it is statistically possible to construct the hybrid model as defined by Eq. (21).

(21) $$Hybride=\frac{1}{2}GM\left(1,1\right)+\frac{1}{2}VAR\left(1\right)$$

**Fig. 1:**
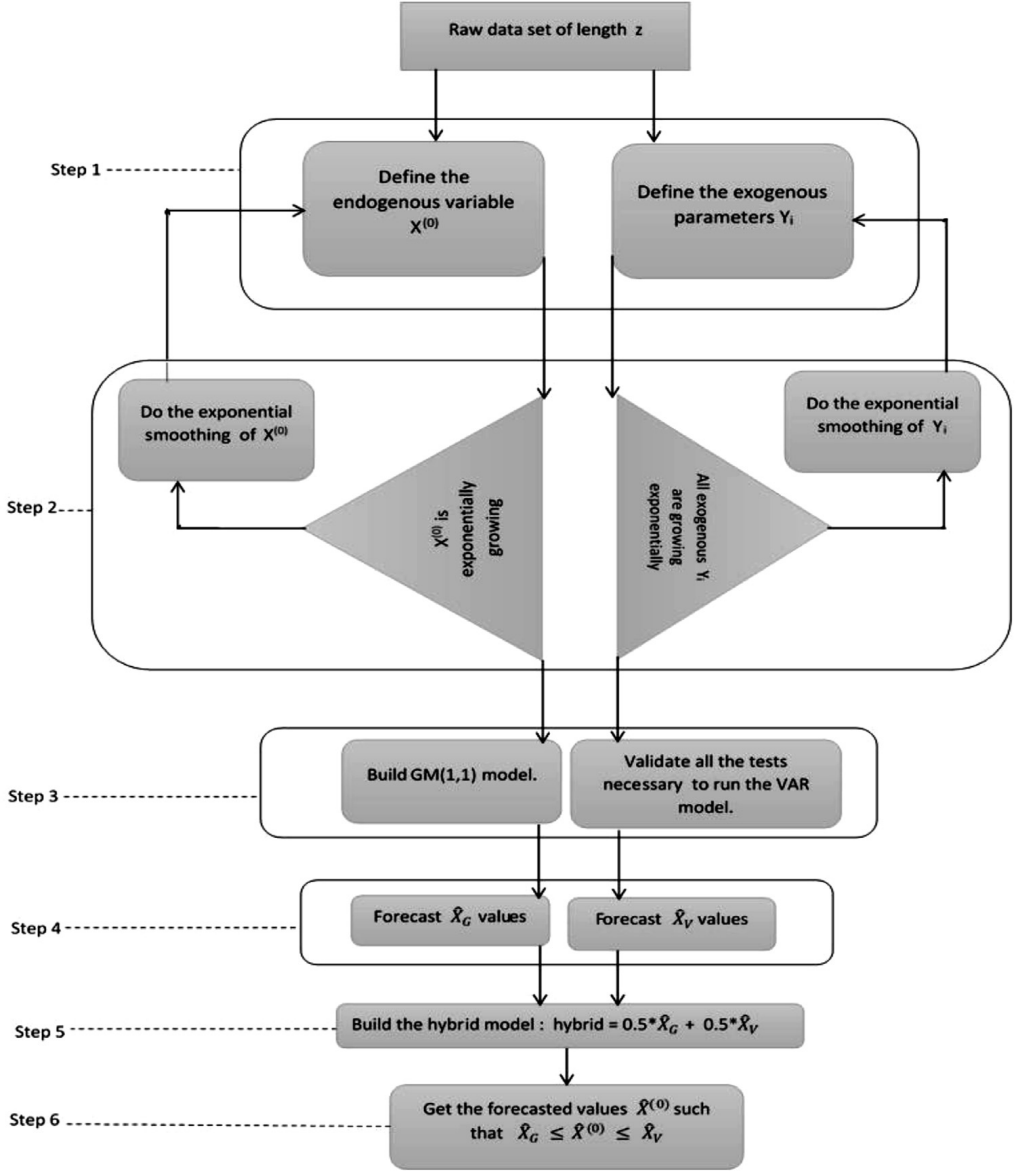
Flow chart of the hybrid model.

This model may be sensitive to moderate fluctuations of electricity consumption. The following flow chart in Fig. 1 shows the key stages that are useful for the execution of the GM(1,1)-VAR(p) model.

The initialization stage requires using all entering data with *z* length.

- **Step 1** requires the designation of the endogenous variables (dependent), as well as that of all exogenous variables(independent). Within the framework of this analysis, X^(0)^ is the endogenous parameter, while the GDP, the GDP per capita, the population, the number of households, and the number of subscribers represent all the exogeneous parameters designated by *Y_i_*.
- Moreover, **step 2** suggests making sure that the exponential growth trend of all variables is effective. In fact, Yuan et al. [1] have proved in 2016, that the GM(1,1) model makes it possible to obtain better forecast- ting results than when entering energy consumption data show an exponential growth trend. This idea is kept in this work as a sine qua non condition to executing the hybrid model. All retained parameters must have an exponential growth trend.

If not, an exponential smooth of entering data will be necessary, and the after-smoothing such as variables will be considered. The authors recommend retain the smooth values whose gap with actual data is below 0.5 [1].

Once the exponential trend of parameters is established,

- **Step 3** recommends building GM(1,1) and VAR(p) models respectively, following the steps described in the previous sections (1) and (2).
- **Step 4** recommends executing each model, and filtering the *X*^ˆ^*_G_* of GM(1,1), and *X*^ˆ^*_V_* of VAR(p).
- **Step 5** recommends building the hybrid model in such a way that
  $$Hybrid=0.5\hat{X}G\;+0.5\hat{X}V.$$
- **Step 6** which is the last, recommends displaying estimated data *X*^ˆ(0)^ in such a way that
  $$\hat{X}G\;\leq \hat{X}\left(0\right)\;\leq 0.5\hat{X}V.$$

## Accuracy of the hybrid model

Table 2 allows a comparison between our results and those obtained by the authors of the hybrid models that are similar to the GM(1,1)-VAR(1) model. Considering their respective MAPEs, one can conclude that the new hybrid model presented in this paper offers a better accuracy and more reliable forecast capacities. Those interesting results are due to the fact that, the new hybrid model includes additional determinants of the demand, which are all primarily characterized by an exponential growth trend. This trend improves the forecasting quality of the model.

**Table 2:** Accuracy of similar hybrid models.

Models	Mapes	Authors	Years
GM(1,1)-ARMA(2,1)	4.39%	Xu W. et al. [6]	2015
GM(1,1)-ARIMA(2,1)	2.30%	Yuan C. et al. [1]	2016
GM(1,1)-VAR(1)	1.63%	Writers	2020

**Fig. 2:**
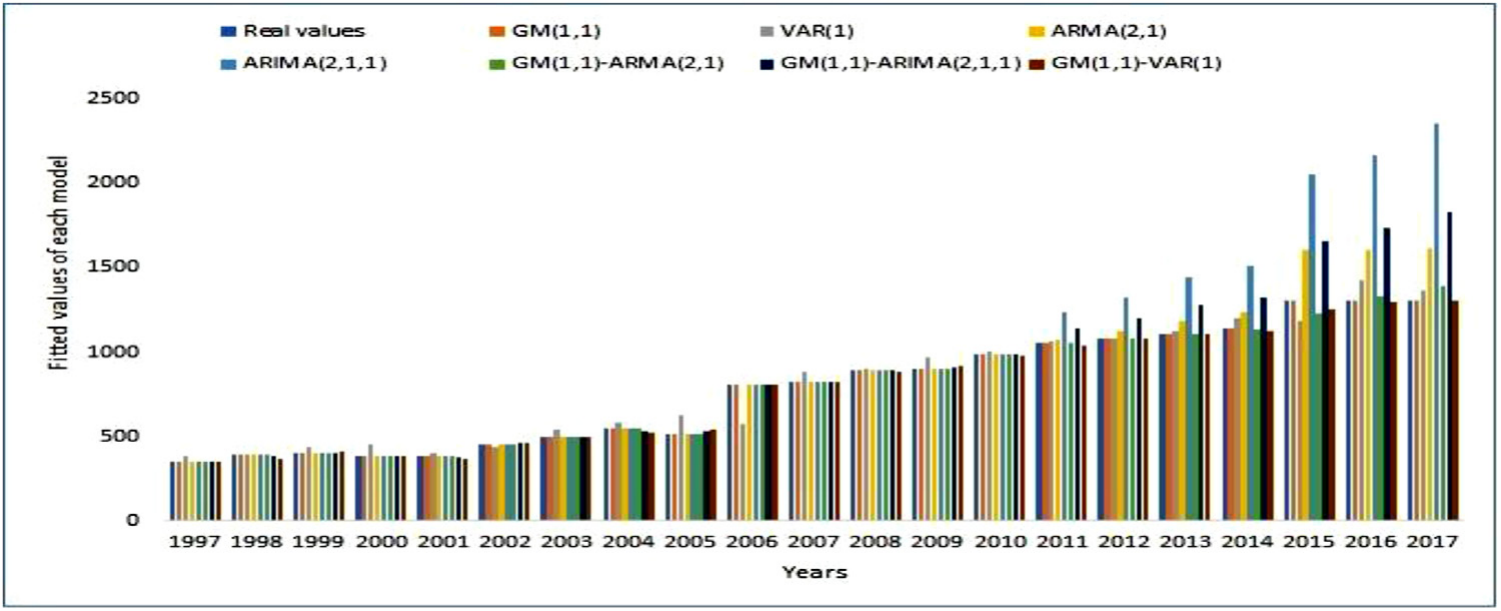
Comparison of the forecast capacities of the various models.

Fig. 2 presents a comparison of the provisional capacities of the GM(1,1)-VAR(1) model, with the capacities of six other models which were reproduced and tested based on our data. It can be observed that over each year, the evolution of data filtered by the GM(1,1)-VAR(1) model on top right, and which is indicated by a brown vertical line is, in most cases, identical to the evolution of actual data regarding electricity consumption of each set of lines, over the 1997–2017 period. The new formulated model surpasses hybrid models of the same nature like GM(1,1)-ARMA(2,1) (in green), and the GM(1,1)- ARIMA(2,1,1) (in dark blue). Therefore, introducing additional determinants of the endogenous variable in such type of model fairly improves their accuracy.

Additional tests and comparisons have allowed us to observe that the new hybrid model can live up with some recent artificial intelligence models. Table 3 allows us to notice that, regarding the values of the various accuracy indicators, the new model is as reliable as hybrid models: the VMD-EELM (Variational Mode Decomposition- Evolutionary Extreme Learning Machine), VMD-SVM- PSO (the VMD which is coupled to the Support Vector Machine and optimized by the PSO algorithm), and VMD-SR-SVRCBCS(the VMD which is coupled to Self-recurrent mechanism and to Support Vector Regression and improved by the CBCS algorithm), recently developed to perform water-flow rate forecasts of hydroelectrical dams [7,8], and electrical loads in China [9]. Type GM(1,N)-VAR(p) hybrid models, whose genesis is given in this paper (for *N* = *p* = 1), could prove to be as efficient as complex artificial intelligence models. Therefore, they can be further developed and tested within the annual forecasting rates of hydroelectrical dams, peak loads on electrical networks, and electricity demand at various stages, depending on the determinants with exponential growth.

**Table 3:** Comparison with recent artificial intelligence hybrid models.

Models	APE	MAPE(%)	RMSE	NS	Authors
VMD-EELM	–	2.960	471.781	–	Niu et al.2020
VMD-SVM-PSO	–	–	3648.830	0.926	Feng et al. 2020
VMD-SR-SVR-CBCS	62.3	0.9	85.5	–	Zhang et al. 2020
GM(1,1)-VAR(1)	1.5	1.629	15.42	0.998	Writers

## Declaration of Competing Interest

The authors declare that they have no known competing financial interests or personal relationships that could have appeared to influence the work reported in this paper.

## Appendix Supplementary materials

Image, application 1

Image, application 2

Image, application 3
